# Diversity and Global Distribution of IncL/M Plasmids Enabling Horizontal Dissemination of *β*-Lactam Resistance Genes among the Enterobacteriaceae

**DOI:** 10.1155/2015/414681

**Published:** 2015-07-08

**Authors:** Marcin Adamczuk, Piotr Zaleski, Lukasz Dziewit, Renata Wolinowska, Marta Nieckarz, Pawel Wawrzyniak, Piotr Kieryl, Andrzej Plucienniczak, Dariusz Bartosik

**Affiliations:** ^1^Department of Bacterial Genetics, Institute of Microbiology, Faculty of Biology, University of Warsaw, Miecznikowa 1, 02-096 Warsaw, Poland; ^2^Institute of Biotechnology and Antibiotics, Staroscinska 5, 02-516 Warsaw, Poland; ^3^Department of Pharmaceutical Microbiology, Medical University of Warsaw, Oczki 3, 02-007 Warsaw, Poland

## Abstract

Antibiotic resistance determinants are frequently associated with plasmids and other mobile genetic elements, which simplifies their horizontal transmission. Several groups of plasmids (including replicons of the IncL/M incompatibility group) were found to play an important role in the dissemination of resistance genes encoding *β*-lactamases. The IncL/M plasmids are large, broad host range, and self-transmissible replicons. We have identified and characterized two novel members of this group: pARM26 (isolated from bacteria inhabiting activated sludge from a wastewater treatment plant) and pIGT15 (originating from a clinical strain of *Escherichia coli*). This instigated a detailed comparative analysis of all available sequences of IncL/M plasmids encoding *β*-lactamases. The core genome of these plasmids is comprised of 20 genes with conserved synteny. Phylogenetic analyses of these core genes allowed clustering of the plasmids into four separate groups, which reflect their antibiotic resistance profiles. Examination of the biogeography of the IncL/M plasmids revealed that they are most frequently found in bacteria of the family Enterobacteriaceae originating from the Mediterranean region and Western Europe and that they are able to persist in various ecological niches even in the absence of direct antibiotic selection pressure.

## 1. Introduction

The discovery of antibiotics initiated a new era in medicine. However, in recent years, the rapidly growing antibiotic resistance of pathogenic bacterial strains has greatly hindered the fight against infectious diseases. This problem is particularly serious in relation to the use of *β*-lactam antibiotics due to the profusion and diversity of *β*-lactamases. The production of these enzymes by Gram-negative bacteria is the most important factor undermining the efficacy of treatment using this group of antibiotics [1].

Two major schemes are used to classify *β*-lactamases: molecular discrimination (proposed by Ambler) based on amino acid sequences and functional discrimination (proposed by Bush-Jacoby-Medeiros) dividing enzymes according to their substrate and inhibitor specificities [2]. The Ambler classification is more commonly used, with each *β*-lactamase class grouping proteins according to discernible features such as molecular weight or conserved amino acid motifs [3].

Ambler classes A, C, and D *β*-lactamases contain a serine residue in their active site, whereas class B includes metalloenzymes (MBLs: metallo-*β*-lactamases) that require zinc ions for substrate hydrolysis. Attempts have been made to assign specific substrates to *β*-lactamases of particular classes (e.g., class A as penicillinases), but this division has proven to be inaccurate as novel enzymes with modified activity profiles are discovered following the introduction of newly developed drugs into clinical practice. For example, a single amino acid alteration in the narrow-spectrum *β*-lactamase SHV-1 may lead to the emergence of enzymes that can hydrolyze extended-spectrum cephalosporins or even carbapenems [4].

The dissemination of *β*-lactamase genes and other antibiotic resistance determinants is a consequence of inter- and intraspecies DNA exchange, occurring mainly due to the transfer of bacterial plasmids. Plasmids are extrachromosomal DNA molecules capable of autonomous replication, which are the major players in horizontal gene transfer and are responsible for shaping bacterial resistomes. A harmless environmental strain may be transformed into a pathogen by the acquisition of a multiresistance plasmid [5].

Plasmids promote the horizontal transfer of antibiotic resistance genes among bacteria belonging to various species or genera. The spread of these genes depends on the host range and conjugative properties of plasmids. Thus, conjugative, broad host range replicons have the greatest impact on overall antibiotic resistance transmission [6, 7].

Amongst broad host range conjugative plasmids, special attention has been paid to IncL/M replicons, one of the six major resistance plasmid groups identified in clinically relevant Enterobacteriaceae [8, 9]. Plasmids representing this incompatibility group have been identified as carriers of various *β*-lactam resistance genes encoding extended-spectrum *β*-lactamases (ESBL), classes A, B, and D carbapenemases, and AmpC *β*-lactamases [10–21]. Plasmid pEL60 of* Erwinia amylovora* was recognized as an archetype for the IncL/M group. It lacks mobile elements and drug resistance genes, typical for other multiresistant IncL/M plasmids [22]. However, a detailed genomic analysis revealed the presence of backbone genes typical for all IncL/M replicons, including replication and stabilization modules, a conjugative transfer system and a* mucAB*-like mutagenic DNA repair system, which is closely related to the* rulAB* operon conferring tolerance to UV radiation [22].

This study comprises an analysis of the complete nucleotide sequences of two SHV-5 *β*-lactamase-encoding plasmids (pARM26 and pIGT15) isolated from environmental and clinical samples, plus a detailed meta-analysis of available sequence data describing IncL/M replicons carrying *β*-lactamase genes.

## 2. Materials and Methods

### 2.1. Bacterial Strains and Culture Conditions

*Escherichia coli* strains DH5*α* [23] and CZD1527 [24] were grown in lysogeny broth (LB) medium [25] at 30°C or 37°C. Plates of solidified LB medium were prepared by the addition of 1.5% agar. Where necessary, the medium was supplemented with ampicillin (100 *μ*g/mL) and/or tetracycline (20 *μ*g/mL).

### 2.2. Isolation of Plasmids

Plasmid pARM26 was isolated exogenously in 2011. A flask containing 19 mL of LB medium supplemented with ampicillin (100 *μ*g/mL) was inoculated with 1 mL of return activated sludge from the “Czajka” wastewater treatment plant (Warsaw, Poland) and incubated overnight at 30°C with aeration. The bacterial growth was pelleted by centrifugation and plasmid DNA isolated by alkaline lysis.* E. coli* DH5*α* was transformed with the purified DNA, selecting on LB agar plates supplemented with ampicillin (100 *μ*g/mL) and tetracycline (20 *μ*g/mL).

Plasmid pIGT15 was isolated in 2005 from the multiresistant and multireplicon* E. coli* strain CZD1527 obtained from a child hospitalized in The Children's Memorial Health Institute in Warsaw (Poland) during the course of standard microbiological diagnostic testing.

### 2.3. DNA Sequencing

The complete nucleotide sequences of plasmids pARM26 and pIGT15 were determined using MiSeq sequencers (Illumina Inc.) in the DNA Sequencing and Oligonucleotide Synthesis Laboratory (oligo.pl) at the Institute of Biochemistry and Biophysics, Polish Academy of Sciences (Warsaw, Poland) and StarSEQ (Mainz, Germany), respectively. After quality filtering, the sequence data were assembled into contigs and scaffolds using Newbler De Novo Assembler v3.0 (Roche). Gaps were closed by the PCR amplification of DNA fragments, followed by Sanger sequencing with an ABI3730xl Genetic Analyzer (Life Technologies) using BigDye Terminator Mix v. 3.1 chemistry (Life Technologies).

### 2.4. DNA Manipulations and the Transformation of Bacterial Cells with Plasmids

The isolation of plasmids and common DNA manipulation methods were carried out as described previously [25]. PCR was performed in a Mastercycler (Eppendorf) using Taq DNA polymerase (Qiagen; with the supplied buffer), dNTP mixture, and appropriate primer pairs. Transformation of* E. coli* strains was performed according to the method of Kushner [28].

### 2.5. Bioinformatics

The plasmid nucleotide sequences were analyzed using Clone Manager (Sci-Ed8) and Artemis [29]. Similarity searches were performed using the BLAST programs [30]. Identified insertion sequences were compared with the ISfinder database [31]. For comparative analyses of plasmid sequences, the Easyfig program was used [32]. Phylogenetic analysis of the IncL/M plasmids was performed using the EDGAR tool [33]. To construct the phylogenetic tree, 20 core genes from the 20 IncL/M plasmid genomes were compared. Multiple alignments were created using MUSCLE software [34] and the highly variable regions of these alignments were removed using Gblocks [35]. The remaining sections of the alignments were concatenated into one multiple alignment and used in the construction of a phylogenetic tree with PHYLIP software [36]. The tree was visualized using TreeView version 1.6.6. [37]. Singletons and the core genes, initially defined using the EDGAR tool, were manually verified with BLASTn.

### 2.6. Nucleotide Sequence Accession Numbers

The sequences of plasmids pARM26 and pIGT15 determined in this study have been deposited in GenBank (NCBI) with the accession numbers KP294350 and KP294351, respectively.

## 3. Results and Discussion

### 3.1. The Structure of Plasmids pARM26 and pIGT15

Two novel antibiotic resistance plasmids belonging to the IncL/M incompatibility group were isolated: pARM26 from bacteria inhabiting activated sludge and pIGT15 from a clinical* E. coli* isolate. DNA sequencing demonstrated that both pARM26 (86,948 bp) and pIGT15 (74,839 bp) are circular plasmids with mean GC contents of 54.4% and 54.7%, respectively.* In silico* sequence analyses revealed that the plasmids carry 107 (pARM26) and 88 (pIGT15) putative genes. A summary of these genes, including their position, transcriptional orientation, the size of the encoded proteins, and their closest known homologs, is presented in Table S1 (Supplementary Materials, available online at http://dx.doi.org/10.1155/2015/414681).

**Figure 1:**
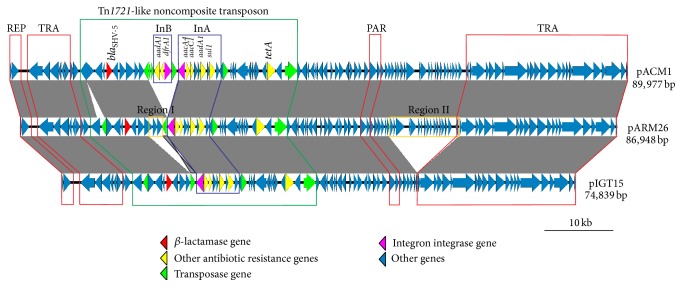
Linear map showing the genetic structure of circular plasmids pACM1, pARM26, and pIGT15. Arrows indicate the direction of gene transcription. The gray-shaded area connects DNA regions of different plasmids with at least 99% nucleotide sequence identity. Yellow frames indicate DNA regions (I and II) of pARM26 not present within pIGT15. REP: replication system; TRA: conjugal transfer system; PAR: partitioning system; InA and InB: class 1 integrons.

Comparative analyses of the plasmid genomes revealed high levels of synteny and sequence identity. Homologs of all the pIGT15 genes are also present in the pARM26 genome and the aligned nucleotide sequences show 99% identity. Plasmid pARM26 is approximately 12 kb larger than pIGT15 because it possesses two additional DNA regions named region I and region II (Figure 1). Region I (nucleotide position 18,704–20,614) contains genes encoding (i) a conserved membrane protein (pIGT15 carries a truncated homologous gene encoding only the N-terminal part of this protein), (ii) a putative L-fuculose-phosphate aldolase (EC 4.1.2.17; which can also function as a D-ribulose-phosphate aldolase) involved in the utilization of L-fucose and D-arabinose [38], and (iii) a hydroxypyruvate isomerase (EC 5.3.1.22, which catalyzes the reversible isomerization of hydroxypyruvate and tartronate semialdehyde [39]). The region II (nucleotide position 53,618–63,898) carries 17 genes encoding (i) a DNA repair protein RadC, (ii) a HTH_XRE transcriptional regulator, (iii) gene expression modulator RmoA, (iv) antirestriction protein KlcA, (v) a mobilization protein C, and (vi) 12 hypothetical proteins of unknown function.

Comparison with the GenBank database revealed that pARM26 and pIGT15 are most closely related to plasmid pACM1 of* Klebsiella oxytoca* [10]. As shown in Figure 1, the three plasmids share gene synteny and show high levels of sequence conservation. Plasmid pACM1, the largest of the replicons, has an additional unique DNA region containing a class I integron carrying two antibiotic resistance gene cassettes (Figure 1). Each of the plasmids also contains several transposable elements (TEs), including (i) the insertion sequences IS*26* (2 copies; some in different genomic locations) and IS*6100* (1 copy), both classified within the IS*6* family [31] and (ii) a large Tn*1721*-like noncomposite Tn*3* family transposon (of 28,815 bp in pARM26, 26,985 bp in pIGT15, and 31,842 bp in pACM1), which is absent in other members of the IncL/M group. These three closely related plasmids are self-transmissible replicons conferring resistance to multiple antibiotics, including aminoglycosides (*aacA4*,* aacC1*, and* aadA1*), trimethoprim (*dfrA1*, only in pACM1), sulfonamides (*sul1*), and tetracycline (*tetA*). They also encode an extended-spectrum *β*-lactamase (ESBL) belonging to the SHV family, SHV-5 (Figure 1). Interestingly, all of the resistance determinants are embedded within the aforementioned Tn*3* family transposon (Figure 1).

It is important to mention that the bacterial host strains carrying these plasmids originated from different environments (i.e., nosocomial and nonnosocomial) and were isolated at different times. The plasmid pACM1 was isolated in 1993 from a cephalosporin-resistant* K. oxytoca* strain from the USA [40], pIGT15 in 2005 from a Polish clinical strain of* E. coli*, and pARM26 in 2011 from bacteria inhabiting a wastewater treatment plant, also in Poland. Comparative genomic analyses of other IncL/M replicons may shed light on the phylogeny and evolution of this group of antibiotic resistance plasmids, which play an important role in the dissemination of *β*-lactamase genes among members of the family Enterobacteriaceae.

### 3.2. Diversity and Comparative Genomics of the IncL/M Plasmids

To our knowledge, until now (December 2014), only eighteen complete nucleotide sequences of IncL/M plasmids have been deposited in public databases (Table 1). With the addition of the two replicons described in this study (pARM26 and pIGT15) the sequences of 20 plasmids from this group were available for comparative genomic analyses.

Comparative analyses revealed that the pan-genome of the IncL/M plasmids consists of 248 genes. This is a relatively small number considering the fact that each of these large plasmids (of 60–90 kb in size; Table 1) carries about 100 genes. This finding indicates that the IncL/M plasmids are highly conserved, sharing the majority of their genes. It is also in accordance with the small number of unique genes (56) identified in the plasmid genomes. Only half of the analyzed IncL/M plasmids contain singletons (Table 2) and their number ranges from 0 to 15 per replicon. The highest number of singleton genes was found in plasmid R830b of* Serratia marcescens*, which does not carry any AR determinants. Several of these genes may play an important role in adaptation of the host strain to changing environmental conditions. Some appear to confer resistance to toxic heavy metals (mercury, cobalt, zinc, and cadmium), while others are probably engaged in formaldehyde detoxification (Table 2). Heavy metal resistance genes (an arsenic resistance gene cluster) were also identified in pKOI-34, another member of the IncL/M group (Table 2). The pool of singletons also includes 11 (19.6%) antibiotic resistance genes (*aadA2*, *bla*_CTX-M-14_, *bla*_FOX-7_, *bla*_IMP-4_, *bla*_IMP-34_, *bla*_KPC-4_,* dfrA1*,* dfrA12*,* mphA*,* strA*, and* strB*), frequently associated with transposable elements (Table 2). This finding is in agreement with previous observations suggesting that the majority of AR genes of the IncL/M plasmids have been acquired within TEs (or mobile integron gene cassettes) inserted at “hotspot” sites in the highly conserved plasmid backbone [10].

**Figure 2:**
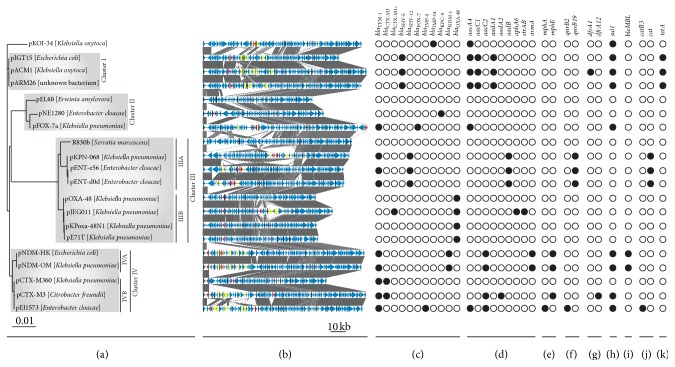
Comparative analyses of IncL/M plasmids. (a) Phylogenetic tree based on the 20 core genes of the IncL/M plasmids. (b) Comparative genomic analysis of the IncL/M plasmids (sequence annotations of several plasmids have been manually corrected; the distinguished genes are shown by arrows on the linear maps: red:* bla* genes; yellow: other AR genes). The gray-shaded areas connect DNA regions of different plasmids with at least 68% nucleotide sequence identity. (c–k) Comparison of the antibiotic resistance gene contents of the IncL/M plasmids: (c) *β*-lactam resistance genes; (d) aminoglycoside resistance genes; (e) macrolide resistance genes; (f) quinolone resistance genes; (g) trimethoprim resistance genes; (h) sulfonamide resistance genes; (i) bleomycin resistance genes; (j) chloramphenicol resistance genes; (k) tetracycline resistance genes.

The core genome of the IncL/M plasmids consists of 20 genes, conserved in all the analyzed replicons. Functional classification of these genes revealed that they encode proteins involved in plasmid replication (*repA, repC*), stable plasmid maintenance (*resD*,* parA*), conjugal transfer (*trbA*,* trbB*,* trbC*,* nikA*,* mobA*,* traH*,* traI*,* traJ*,* traM*,* traN*,* traO*,* traQ*,* traU*,* traW*, and* traY*), and UV resistance (*mucA*). In an effort to understand the evolution of these plasmids, a phylogenetic analysis was performed based on the amino acid sequences of the proteins encoded by the core genes (Figure 2). Within the constructed phylogenetic tree, four main clusters are evident, grouping closely related replicons.

The first cluster (cluster I) is comprised of the plasmids pARM26 and pIGT15, identified in this work, and also the closely related pACM1. All three are multiresistance replicons carrying 7 (or 8 in the case of pACM1 [10]) AR genes, including a* tetA* gene conferring resistance to tetracycline, which is not present in other IncL/M plasmids (Figure 2). The second cluster (cluster II) contains two multiresistance replicons (pFOX-7a, pNE1280) [15, 20] and plasmid pEL60 of the plant pathogen* E. amylovora*, which does not contain any AR genes [22] (Figure 2). The third and largest cluster (cluster III) is divided into two subclusters. The IIIA branch groups closely related plasmids pKPN-068, pENT-e56, and pENT-d0d, carrying five common, identical AR genes (*bla*_TEM-1_, *bla*_SHV-12_,* aadB*,* qnrB19*, and* cat*) [26], as well as the R830b replicon (containing no AR genes), which forms an outgroup within this subcluster (Figure 2). The IIIB branch gathers four similar plasmids (pOXA-48a, pJEG011, pKPoxa-48N1, and pE71T) with an embedded *bla*_OXA-48_ gene [13, 16, 17, 21]. Interestingly, within this subcluster there is only one multiresistance plasmid, pJEG011 [16] (Figure 2). The fourth cluster (cluster IV) is also divided into two smaller subclusters: IVA, consisting of two plasmids, pNDM-HK and pNDM-OM, with a high level of genetic synteny [18, 19], and IVB, grouping closely related plasmids pCTX-M3, pCTX-M360, and pEl1573 [11, 12, 14] (Figure 2).

The results of this phylogenetic analysis confirmed previous assumptions that there were probably several lineages of the IncL/M plasmids before they began acquiring diverse exogenous segments of DNA [10]. A more detailed comparative analysis showed that the insertions occurred within several “hotspots,” which are usually located between conserved core genes in the plasmid backbone. Insertions at other locations were observed in only a few cases, for example, in the plasmids pOXA-48a, pJEG011, pE71T, and pKPoxa-48N1 (all grouped in the subcluster IIIB), in which the acquired DNA interrupted the* tir* gene encoding a transfer inhibition protein.

### 3.3. IncL/M Plasmids: A Reservoir of *β*-Lactamase Genes

Among the 20 completely sequenced IncL/M plasmids, 18 encode at least one *β*-lactam resistance gene. In total, these plasmids carry 28 genes encoding 8 families of *β*-lactam hydrolyzing enzymes, representing all of the classes distinguished by Ambler: TEM, CTX-M, SHV, and KPC of Ambler class A; IMP and NDM of class B; FOX of class C; and OXA of class D [41, 42].

The most common is the *bla*_TEM-1_ gene (encoding the TEM-1 type enzyme), which is present in 9 plasmids: (i–iv) pFOX-7a, pKPN-068, pENT-e56, and pENT-d0d, representing clusters II and III in the phylogenetic tree (Figure 2), and (v–ix) pNDM-OM, pNDM-HK, pCTX-M3, pCTX-M360, and pEl1573, all belonging to cluster IV (Figure 2). Interestingly, all of the aforementioned plasmids also encode another *β*-lactamase inactivating a wider range of *β*-lactams. The presence of this second *β*-lactamase gene reflects the relatively narrow substrate specificity of the TEM-1 type enzyme, which is capable of hydrolyzing only penicillins and narrow-spectrum cephalosporins (such as cephalothin or cefazolin), but not more recently developed higher-generation cephalosporins with an oxyimino side chain (oxyimino-cephalosporins) [2]. Thus, the resistance conferred by the TEM-1 enzyme (probably the most common among enteric bacteria [43]) is in fact currently ineffective and has been “bolstered” by the acquisition of other genes encoding *β*-lactamases with a broader specificity in the course of IncL/M plasmid evolution.

The IncL/M plasmids also frequently encode *β*-lactam hydrolyzing enzymes of the SHV family, which gathers more than 160 *β*-lactamases [44], including the common enzymes SHV-5 and SHV-12 [45]. The *bla*_SHV-5_ genes are carried by three closely related plasmids, pARM26, pIGT15, and pACM1 (cluster I in the phylogenetic tree), while *bla*_SHV-12_ genes are present within plasmids pKPN-068, pENT-e56, and pENT-d0d of the subcluster IIIA (Figure 2).

The IncL/M plasmids also encode enzymes representing the CTX-M family of ESBLs. These enzymes are less closely related to TEM or SHV *β*-lactamases but show high similarity to the *β*-lactamases of various* Kluyvera* species [46]. Three of the IncL/M plasmids encode the CTX-M family *β*-lactamases: (i-ii) pCTX-M3 and pCTX-M360 (*bla*_CTX-M3_) and (iii) pJEG011 (*bla*_CTX-M14_) (Figure 2).

Another class of *β*-lactamases encoded by the IncL/M plasmids is represented by KPC-4, a carbapenemase of the KPC family. The gene encoding this enzyme is located within the transposon Tn*4401f* embedded in the plasmid pNE1280 [20].

Several IncL/M plasmids also encode metallo-*β*-lactamases (MBL) of Ambler class B, that is, enzymes that require metal ions as cofactors for their activity [47]. MBLs are the most clinically important carbapenemases due to their ability to hydrolyze almost all *β*-lactams, except monobactams. Moreover, they are not susceptible to therapeutic *β*-lactam inhibitors, for example, clavulanate [48]. MBL genes were found within (i) pEl1573 (*bla*_IMP-4_ gene), (ii) pKOI-34 (*bla*_IMP-34_), and (iii-iv) two nearly identical plasmids of the subcluster IVA, pNDM-HK and pNDM-OM (*bla*_NDM-1_) (Figure 2). It is important to note that the *bla*_IMP-4_ and *bla*_IMP-34_ genes of pEl1573 and pKOI-34 are in fact class 1 integron gene cassettes, which is typical for most, if not all, genes encoding IMP-, VIM-, and GIM-type MBLs [47].

An Ambler class C *β*-lactamase (AmpC-type, CBL) is encoded by only one IncL/M plasmid, pFOX-7a, which was isolated from strains of* Klebsiella pneumoniae* associated with an outbreak in a neonatal intensive care unit in central Italy [15]. The FOX-7 enzyme can hydrolyze extended-spectrum cephalosporins (e.g., cephamycins) and is resistant to various combinations of *β*-lactamase inhibitors [15]. The *bla*_FOX-7_ gene is unique in the current pan-genome of the IncL/M plasmids (Figure 2, Table 2).

Four of the IncL/M plasmids (pOXA-48, pJEG011, pKPoxa-48N1, and pE71T) (subcluster IIIB; Figure 2) carry the *bla*_OXA-48_ gene encoding a *β*-lactamase of Ambler class D. This enzyme is an OXA-family carbapenemase, which can hydrolyze penicillins and carbapenems but not extended-spectrum cephalosporins [21].

Epidemiological surveys have revealed that IncL/M plasmids play an important role in the global dissemination of *β*-lactam resistance genes (e.g., [49]). These replicons are not simply carriers of these resistance genes; they may also be considered “donors” of* bla *genes to other replicons coresiding in a particular host.

Close inspection of the IncL/M plasmid genomes revealed that the majority of their *β*-lactamase genes are associated with transposable elements (e.g., (i) *bla*_TEM-1_ is present within Tn*2* of pCTX-M360, pCTX-M3, pNDM-HK, pNDM-OM, and pEl1573, (ii) *bla*_CTX-M3_ was mobilized by IS*Ecp1* into pCTX-M3 and pCTX-M360, and (iii) *bla*_OXA-48_ occurs in Tn*1999* of pOXA-48a) or they persist in the form of integron gene cassettes (*bla*_IMP-4_ of pEl1573 and *bla*_IMP-34_ of pKOI-34). This localization significantly enhances the “mobility” of the resistance genes, which can be readily transferred from the IncL/M plasmids into bacterial chromosomes, as shown for the *bla*_OXA-48_ gene associated with the IS*1R*-based composite transposon Tn*6237* [50]. However, an even greater epidemiological threat is posed by the transfer of* bla* genes into other plasmids coresiding in a bacterial cell. This may result in the generation of novel resistance plasmids with a host range that is different from that of the parental IncL/M plasmids. Therefore, plasmid-mediated gene transfer can play a fundamental role in the crossing of taxonomic boundaries and may significantly influence the prevalence of *β*-lactam resistance.

It is notable that 13 of the analyzed IncL/M plasmids also contain other AR genes, which confer resistance to antibiotics representing 8 drug classes: aminoglycosides, macrolides, quinolones, trimethoprim, sulfonamides, bleomycin, chloramphenicol, and tetracycline (Figure 2). Interestingly, this phenomenon of multiresistance has also been observed in several other types of plasmids encoding extended-spectrum *β*-lactamases (e.g., [51]). In consequence, the choice of appropriate antibiotics to eliminate *β*-lactamase-producing pathogenic bacterial strains is becoming more and more limited [52].

### 3.4. Biogeography of the IncL/M Plasmids

**Figure 3:**
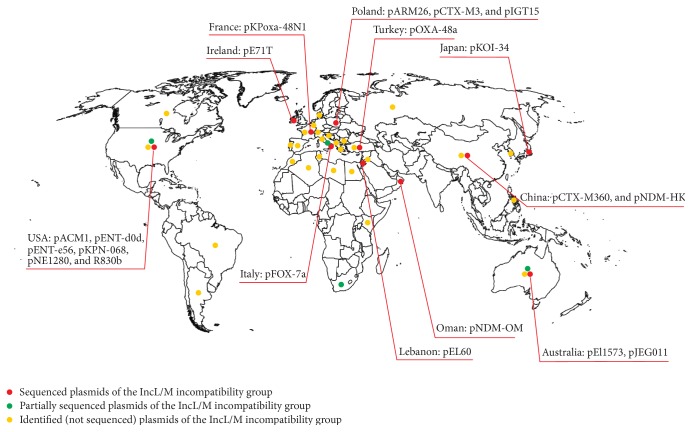
Geographical distribution of IncL/M plasmids.

Although IncL/M broad host range, self-transferable plasmids play an important role in the horizontal transmission of multiresistance phenotypes among Enterobacteriaceae, still very little is known about the diversity and distribution of these replicons in different environments. As mentioned above, the genetic structure of only 20 replicons of this group has been revealed to date. However, database searches (performed at the NCBI website) produced some information about four other members of the IncL/M group that have been partially sequenced and subjected to molecular analyses. These are R446b of* Morganella morganii* [53], R471a of* Serratia marcescens* [54], pMU407.1 of* Klebsiella pneumoniae* [55], and p61/9 of* Salmonella enterica* serovar Typhimurium [56]. Moreover, in depth analysis of sequence accessions in the NCBI database yielded further information concerning IncL/M plasmids (usually identified by PCR-based replicon typing) in samples originating from various environments. Most of the detected IncL/M plasmids were carried by pathogenic strains of Enterobacteriaceae isolated from hospitalized patients. These strains originated from 27 countries: Albania [57], Algeria [58], Argentina [59], Australia [60], Belgium [61], Brazil [62], Bulgaria [63], Canada [64], China [65], Croatia [66], Denmark [67], Egypt [21], France [68], Greece [69], Kenya [70], Korea [71], Lebanon [21], Libya [72], Morocco [21], Philippines [69], Portugal [73], Russia [74], Spain [75], Switzerland [76], Tunisia [77], Turkey [21], and USA [78]. IncL/M plasmids were also found in enterobacteria isolated from companion animals in Italy [79] and from an urban wastewater treatment plant in Algeria [80]. Thus, IncL/M plasmids have so far been identified in 31 countries on 6 continents, which points to their global distribution (Figure 3). Notably, the majority of strains carrying the IncL/M plasmids were identified in countries located near the Mediterranean Sea and in Western Europe (Figure 3).

Majority of the IncL/M plasmids were isolated from pathogenic strains of Enterobacteriaceae in the course of epidemiological studies. The unique genes found within these plasmids are usually involved in antibiotic resistance (Table 2) and may be linked with the specific medical treatment in the particular health care unit. This exemplifies the role of mobile genetic elements in transfer of genes, enabling bacteria to improve their fitness under local stress conditions [81]. Such phenomenon is common for environmental bacterial strains, which are able to survive under various harsh conditions (e.g., [82–84]). This study illustrates that the IncL/M plasmids are not exclusively associated with pathogenic enterobacteria but are also found in nonnosocomial environments, including communal wastes. Wide dissemination of these multiresistance plasmids and their possible transfer into pathogenic bacteria make them an important and considerable epidemiological threat.

## 4. Conclusions

The plasmids of the IncL/M incompatibility group are broad host range, multiresistance, self-transmissible replicons, which actively participate in the horizontal transfer of antibiotic resistance genes. These replicons have been associated with several outbreaks of disease caused by pathogenic strains of Enterobacteriaceae all over the world. They are also involved in the dissemination of *β*-lactam resistance, since they carry genes encoding enzymes representing all four Ambler classes of *β*-lactamases: Class A: TEM-1, SHV-5, SHV-12, CTX-M3, CTX-M14, and KPC-4; Class B: IMP-4, IMP-34, and NDM-1; Class C: FOX-7; and Class D: OXA-48.

Detailed analysis of the IncL/M plasmid genomes revealed that they have highly conserved backbones and share significant gene synteny. Moreover, phylogenetic analysis showed that these replicons may be grouped into four clusters, which also reflect the distribution of the antibiotic resistance determinants.

This study is the first to present available data indicating the global dissemination of bacteria carrying IncL/M plasmids. These multiresistance replicons not only are maintained by pathogenic strains of Enterobacteriaceae but also have been detected in bacteria inhabiting different nonnosocomial environments. A good example is the *β*-lactamase-encoding plasmid pARM26 characterized in this study, which was isolated from a wastewater treatment plant in Poland. Its identification strongly suggests that IncL/M plasmids have the ability to persist in various ecological niches even in the absence of direct antibiotic selection. Therefore, these plasmids constitute a rich reservoir of AR genes that may be disseminated among diverse bacteria (including pathogenic strains) in the natural environment.

## Supplementary Material

Table S1. Genes located within plasmids pARM26 and pIGT15.

## Figures and Tables

**Table 1:** IncL/M plasmid sequences deposited in the GenBank database.

Plasmid name	Host strain	Strain origin	Size (bp)	% GC	Reference/GenBank accession number
pACM1	*Klebsiella oxytoca *	New York, USA; hospital patient	89,977	54.4	[10]/NC_024997
pARM26	unknown bacterium	Warsaw, Poland; wastewater treatment plant	86,948	54.4	this work/KP294350
pCTX-M3	*Citrobacter freundii *2526	Warsaw, Poland; hospital patient	89,468	51.0	[11]/NC_004464
pCTX-M360	*Klebsiella pneumoniae* 0773	Guangzhou, China; hospital patient	68,018	51.4	[12]/NC_011641
pE71T	*Klebsiella pneumoniae* E71T	Ireland; hospital patient	63,578	51.2	[13]/NC_023027
pEl1573	*Enterobacter cloacae* El1573	Sydney, Australia; hospital patient	87,731	52.8	[14]/NC_019368
pEL60	*Erwinia amylovora* LebB66	Lebanon; fruits	60,145	51.5	[22]/NC_005246
pENT-d0d	*Enterobacter cloacae* ECNIH5	Bethesda, USA; hospital sink drain	77,575	50.7	[26]/CP009857
pENT-e56	*Enterobacter cloacae* ECNIH4	Bethesda, USA; hospital sink drain	77,248	51.5	[26]/CP009852
pFOX-7a	*Klebsiella pneumonia*e 45A03	Siena, Italy; hospital patient	90,439	53.4	[15]/NC_025134
pIGT15	*Escherichia coli* CZD1527	Warsaw, Poland; hospital patient	74,839	54.7	this work/KP294351
pJEG011	*Klebsiella pneumoniae* Kp002	Sydney, Australia; hospital patient	71,446	50.6	[16]/NC_021078
pKOI-34	*Klebsiella oxytoca* MS5279	Japan; hospital patient	87,343	53.3	[27]/AB715422
pKPN-068	*Klebsiella pneumoniae *KPNIH27	USA; hospital patient	80,411	51.3	—/CP007733
pKPoxa-48N1	*Klebsiella pneumoniae *KP1a	Nancy, France; hospital patient	62,592	51.1	[17]/NC_021488
pNDM-HK	*Escherichia coli* HK-01	Hong Kong, China; hospital patient	88,803	51.5	[18]/NC_019063
pNDM-OM	*Klebsiella pneumoniae* 601	Oman; hospital patient	87,185	51.5	[19]/NC_019889
pNE1280	*Enterobacter cloacae* 1623	Omaha, USA; hospital patient	66,531	52.3	[20]/NC_019346
pOXA-48a	*Klebsiella pneumoniae *Kp11978	Turkey; hospital patient	61,881	51.1	[21]/NC_019154
R830b	*Serratia marcescens *	USA	81,793	53.2	—/NC_019344

**Table 2:** Unique genes identified within IncL/M plasmids.

Plasmid name	Number of singletons	Singleton gene/locus tag [possible function]
pACM1	1	*dfrA1*/D733_p1025 [dihydrofolate reductase]

pARM26	0	—

pCTX-M3	3	*dfrA12*/pCTX-M3_084 [dihydrofolate reductase]; *orfF*/pCTX-M3_085 [integron gene cassette, hypothetical protein]; *aadA2*/pCTX-M3_086 [streptomycin 3′-adenyltransferase]

pCTX-M360	0	—

pE71T	0	—

pEl1573	5	*mphA*/D727_p1024 [macrolide phosphorylase]; *mrx*/D727_p1026 [protein of unknown function, required for MphA expression]; *mphR(A)*/D727_p1027 [transcriptional regulator for macrolide phosphorylase gene *mph(A*)]; *catB3*/D727_p1035 [chloramphenicol acetyltransferase]; *bla*_IMP-4_/D727_p1038 [IMP-4 metallo-*β*-lactamase]

pEL60	2	*orf8*/pEL60p08 [hypothetical protein]; *kfrA*/pEL60p09 [plasmid maintenance protein KfrA]

pENT-d0d	0	—

pENT-e56	0	—

pFOX-7a	9	bla_FOX-7_/D647_p51042 [FOX-7 *β*-lactamase]; *mdrL*/D647_p51043 [major facilitator transporter]; *lysR*/D647_p51044 [LysR family transcriptional regulator];* tnpA*/D647_p51045 [transposase of IS*1634*-family insertion sequence]; tnpA/D647_p51046 [transposase of IS*Apu1* (IS*4* family/IS*H8* group)]; D647_p51047 [hypothetical protein]; *tnpA*/D647_p51048 [transposase of IS*Apu2* (IS*4* family/IS*H8* group)]; *istA*/D647_p51052 [IstA protein of IS*1326* (IS*21* family)]; *istB*/D647_p51053 [IstB protein of IS*1326* (IS*21* family)]

pIGT15	0	—

pJEG011	5	*strA*/D647_p23045 [streptomycin phosphotransferase A]; *strB*/D647_p23040 [streptomycin phosphotransferase B]; *aphA6*/D647_p23043 [aminoglycoside 3′-phosphotransferase]; tnpA/D647_p23042 [transposase of IS*1*-family insertion sequence]; *bla*_CTX-M-14_/D647_p23049 [extended-spectrum *β*-lactamase]

pKOI-34	7	*arsR* [arsenical resistance operon repressor]; arsD [arsenical resistance operon transacting repressor]; *arsA* [arsenical pump-driving ATPase]; *arsB* [arsenic efflux pump protein]; *arsC* [arsenate reductase]; *ycgA* [arsenical pump-driving ATPase]; *bla*_IMP-34_ [IMP-34 metallo-*β*-lactamase]

pKPN-068	0	—

pKPoxa-48N1	0	—

pNDM-HK	1	*insL*/D616_p59011 [transposase of IS*4* family/IS*231* group insertion sequence]

pNDM-OM	0	—

pNE1280	8	*istB*/KPC4_0043 [IstB protein of IS*21*-family insertion sequence]; *bla*_KPC-4_/KPC4_0045 [*β*-lactamase]; *tnpA*/KPC4_0047 [transposase]; KPC4_0049 [hypothetical protein]; KPC4_0051 [hypothetical protein]; KPC4_0053 [hypothetical protein]; *rom*/KPC4_0055 [Rop family protein]; *exc1*/KPC4_0057 [exclusion protein 1]

pOXA-48a	0	—

R830b	15	*tniQ*/D739_p1005 [transposase helper protein TniQ]; D739_p1006 [resolvase/integrase]; D739_p1010 [organomercurial lyase]; D739_p1020 [putative reverse transcriptase]; D739_p1024 [hypothetical protein]; D739_p1025 [Il-IS_2, transposase]; D739_p1026 [lipoprotein signal peptidase]; D739_p1027 [cobalt-zinc-cadmium resistance protein CzcD]; D739_p1028 [Cd(II)/Pb(II)-responsive transcriptional regulator]; D739_p1029 [hypothetical protein]; D739_p1107 [resolvase]; D739_p1108 [S-formylglutathione hydrolase]; D739_p1109 [glyoxalase]; D739_p1110 [S-(hydroxymethyl)glutathione dehydrogenase]; D739_p1111 [uncharacterized protein YaiN in formaldehyde detoxification operon]
