# Supplementary material for: Diversity and Global Distribution of IncL/M Plasmids Enabling Horizontal Dissemination of β-Lactam Resistance Genes among the Enterobacteriaceae
Source: Biomed Res Int. 2015 Jul 8;2015:414681. doi: 10.1155/2015/414681 (PMC4510254; doi:10.1155/2015/414681)
Supplement: Supplementary file 1 — Table S1. Genes located within plasmids pARM26 and pIGT15. [file 414681.f1.pdf]

Supplementary Table 1. Genes located within plasmids pARM26 and pIGT15.

Gene no.	Coding region (bp)	Strand	Protein size (aa)	Possible function	Best BLAST hits		
					aa identity (%)	Organism (plasmid)	GenBank accession no.
				**Plasmid pARM26 (86,948 bp)**			
1	297-1352	→	351	replication initiator protein, RepA	351/351 (100%)	*Enterobacter cloacae* (pENT-e56)	AIX57116
2	2725-4812	←	695	conjugation protein TrbC	695/695 (100%)	*Serratia marcescens* (R830b)	YP_006964547
3	4825-5844	←	339	conjugation protein TrbB	339/339 (100%)	*Klebsiella oxytoca* (pACM1)	YP_009062595
4	5786-7093	←	435	conjugation protein TrbA	435/435 (100%)	*Klebsiella oxytoca* (pACM1)	YP_009062594
5	7093-7488	←	131	conjugation protein TrbN	131/131 (100%)	*Klebsiella oxytoca* (pACM1)	YP_009062593
6	7593-7988	←	131	hypothetical protein	131/131 (100%)	*Klebsiella oxytoca* (pACM1)	YP_009062592
7	8056-8709	→	217	transfer inhibition protein Tir	217/217 (100%)	*Klebsiella oxytoca* (pACM1)	YP_009062591
8	8862-9296	→	144	error-prone repair protein, MucA	144/144 (100%)	*Klebsiella oxytoca* (pACM1)	YP_009062590
9	9245-10090	→	281	translesion synthesis protein, MucB, truncated	280/281 (99%)	*Escherichia coli* HVH 87	EQP96765
10	10279-11676	←	465	methyl-accepting chemotaxis protein	465/465 (100%)	*Escherichia coli* (p1658/97)	NP_862996
11	11849-12553	←	234	IS26 transposase	234/234 (100%)	*Klebsiella pneumoniae* (pNDM-OM)	AFZ84500
12	12578-13495	→	305	galactoside permease	305/305 (100%)	*Escherichia coli* (p1658/97)	NP_863016
13	13526-14614	←	362	recombination protein, RecF	362/362 (100%)	*Klebsiella oxytoca* (pACM1)	YP_009062589
14	14702-14983	←	93	putative K+ transporting ATPase, KdpC subunit	93/93 (100%)	*Klebsiella pneumoniae* subsp. *pneumoniae* KpQ3	EKF78970
15	15260-16120	→	286	SHV-5 beta-lactamase	286/286 (100%)	*Klebsiella oxytoca* (pACM1)	YP_009062588
16	16141-16902	←	253	DeoR family transcriptional regulator	253/253 (100%)	*Providencia stuartii* (pTC2)	YP_006965583
17	17163-18065	→	300	putative dehydrogenase	300/300 (100%)	*Klebsiella oxytoca* (pACM1)	YP_009062586
18	18077-19342	→	421	putative ygbK protein	421/421 (100%)	*Klebsiella oxytoca* (pACM1)	AAL89460.2
19	19335-19955	→	206	putative L-fuculose-1-phosphate aldolase	206/206 (100%)	*Klebsiella oxytoca* (pACM1)	AAL89459
20	19960-20721	→	253	hydroxypyruvate isomerase	253/253 (100%)	*Escherichia coli* (p1658/97)	NP_863008
21	20667-21371	←	234	IS26 transposase	234/234 (100%)	*Klebsiella pneumoniae* (pNDM-OM)	AFZ84500
22	21446-22459	←	337	integrase	337/337 (100%)	*Escherichia coli* (p1658/97)	NP_863006
23	22675-23193	→	172	aminoglycoside 6'-N-acetyltransferase	172/172 (100%)	*Klebsiella oxytoca* (pACM1)	AAC77498
24	23269-23733	→	154	aminoglycoside 3'-acetyltransferase	154/154 (100%)	*Klebsiella oxytoca* (pACM1)	YP_009062574
25	23852-24364	→	170	GCN5 family acetyltransferase	170/170 (100%)	*Klebsiella oxytoca* (pACM1)	YP_009062573
26	24380-24685	→	101	hypothetical protein	101/101 (100%)	*Klebsiella oxytoca* (pACM1)	AAC77502
27	24777- 25568	→	263	3''-O-adenyltransferase	263/263 (100%)	*Klebsiella oxytoca* (pACM1)	YP_009062571
28	25732-26079	→	115	QacE-delta1	115/115 (100%)	*Klebsiella pneumoniae* (pFOX-7a)	YP_009067913
29	26073-26912	→	279	dihydropteroate synthase type I	279/279 (100%)	*Klebsiella oxytoca* (pACM1)	AIM47853
30	27040-27540	→	166	hypothetical protein	166/166 (100%)	*Shigella flexneri 2b*(R100)	NP_052894
31	27509-28018	←	169	NTP-binding protein	169/169 (100%)	*Acinetobacter baumannii* AB0057	YP_002317676
32	28017-28811	→	264	IS6100 transposase	264/264 (100%)	*Trueperella pyogenes*	NP_940743
33	29049-29261	←	70	Urf2Y	70/70 (100%)	*Klebsiella oxytoca* (pACM1)	YP_009062566

Gene no.	Coding region (bp)	Strand	Protein size (aa)	Possible function	Best BLAST hits		
					aa identity (%)	Organism (plasmid)	GenBank accession no.
34	29327-29563	←	78	mercury resistance protein, MerE	78/78 (100%)	*Serratia marcescens* (R478)	NP_941189
35	29560-29925	←	121	transcriptional regulator MerD	121/121 (100%)	*Serratia marcescens* (R478)	NP_941190.2
36	29943-31628	←	561	mercuric reductase, MerA	561/561 (100%)	*Serratia marcescens* (R478)	NP_941191
37	31667-32092	←	141	mercury transport protein, MerC	141/141 (100%)	*Serratia marcescens* (R478)	NP_941192
38	32120-32395	←	91	mercuric transport protein periplasmic component, MerP	91/91 (100%)	*Serratia marcescens* (R478)	NP_941193
39	32411-32776	←	121	mercuric transport protein, MerT	121/121 (100%)	*Serratia marcescens* (R478)	NP_941194
40	32848-33303	→	151	transcriptional regulator, MerR	151/151 (100%)	*Serratia marcescens* (R478)	NP_941195
41	33715-34392	←	225	tetracycline resistance repressor protein, TetR	225/225 (100%)	uncultured bacterium (pRSB101)	YP_133837
42	34396-35670	→	424	tetracycline resistance protein, TetA	424/424 (100%)	uncultured bacterium (pRSB201)	YP_009064288
43	35702-36586	←	294	PecM-like protein	294/294 (100%)	uncultured bacterium (pTB11)	YP_112360
44	37075-38871	→	598	truncated Tn3 transposase	598/598 (100%)	*Klebsiella oxytoca* (pACM1)	AIM47840
45	38932-39369	→	145	translesion synthesis protein, MucB, truncated	145/145 (100%)	*Klebsiella pneumoniae* (pFOX-7a)	YP_009067897
46	39392-40240	←	282	exonuclease	282/282 (100%)	*Klebsiella oxytoca* (pACM1)	YP_009062646
47	40243-40563	←	106	hypothetical protein	106/106 (100%)	*Klebsiella oxytoca* (pACM1)	YP_009062645
48	40708-42346	←	212	hypothetical protein	212/212 (100%)	*Klebsiella oxytoca* (pACM1)	YP_009062644
49	41415-41624	←	69	hypothetical protein	69/69 (100%)	*Citrobacter freundii* (pCTX-M3)	NP_774971
50	41627-41845	←	72	hypothetical protein	72/72 (100%)	*Citrobacter freundii* (pCTX-M3)	NP_774972
51	41890-42573	←	227	hypothetical protein	227/227 (100%)	*Klebsiella oxytoca* (pACM1)	YP_009062643
52	42570-43229	←	219	RelB antitoxin domain-containing protein	219/219 (100%)	*Klebsiella oxytoca* (pACM1)	YP_009062642
53	43732-44037	→	101	hypothetical protein	89/100 (89%)	*Erwinia amylovora* (pEL60)	NP_943209
54	44330-44914	→	194	hypothetical protein	194/194 (100%)	*Klebsiella oxytoca* (pACM1)	YP_009062641
55	44911-45630	→	239	hypothetical protein	239/239 (100%)	*Klebsiella oxytoca* (pACM1)	YP_009062640
56	45791-46339	←	182	hypothetical protein	182/182 (100%)	*Klebsiella oxytoca* (pACM1)	YP_009062639
57	46480-46941	→	153	hypothetical protein	153/153 (100%)	*Klebsiella oxytoca* (pACM1)	YP_009062638
58	46480-46941	→	195	hypothetical protein	195/195 (100%)	*Klebsiella oxytoca* (pACM1)	YP_009062637
59	47763-48248	→	161	hypothetical protein	161/161 (100%)	*Klebsiella oxytoca* (pACM1)	YP_009062636
60	48512-49252	→	246	resolvase	246/246 (100%)	*Erwinia amylovora* (pEL60)	NP_943215
61	49493-50467	→	324	plasmid segregation protein ParM	324/324 (100%)	*Erwinia amylovora* (pEL60)	NP_943216
62	50470-50913	→	147	plasmid stability protein B	147/147 (100%)	*Erwinia amylovora* (pEL60)	NP_943217
63	50923-51474	→	183	endonuclease Nuc	183/183 (100%)	*Erwinia amylovora* (pEL60)	NP_943218
64	51590-52096	→	168	hypothetical protein	168/168 (100%)	*Erwinia amylovora* (pEL60)	NP_943219
65	52089-52568	→	159	hypothetical protein	159/159 (100%)	*Erwinia amylovora* (pEL60)	NP_943220
66	52597-53007	→	136	hypothetical protein	136/136 (100%)	*Serratia marcescens* (R830b)	YP_006964582
67	53125-53388	→	87	hypothetical protein	87/87 (100%)	*Citrobacter freundii* (pCTX-M3)	NP_774992
68	53410-53772	→	120	hypothetical protein	120/120 (100%)	*Erwinia amylovora* (pEL60)	NP_943223
69	53894-54343	→	149	DNA repair protein RadC	149/149 (100%)	*Erwinia amylovora* (pEL60)	NP_943224

Gene no.	Coding region (bp)	Strand	Protein size (aa)	Possible function	Best BLAST hits		
					aa identity (%)	Organism (plasmid)	GenBank accession no.
70	54388-54654	→	88	transcriptional repressor KorC	88/88 (100%)	*Erwinia amylovora* (pEL60)	NP_943225
71	54718-56001	→	427	DGQHR domain-containing protein	427/427 (100%)	*Klebsiella oxytoca* (pACM1)	YP_009062626
72	56609-56788	→	59	CcgAI	59/59 (100%)	*Erwinia amylovora* (pEL60)	NP_943227
73	56795-57721	→	308	hypothetical protein	308/308 (100%)	*Klebsiella oxytoca* (pACM1)	YP_009062625
74	57911-58249	→	112	hypothetical protein	112/112 (100%)	*Enterobacter cloacae ECNIH5* (pENT-d0d)	AIX61617
75	58347-58577	→	76	RmoA	76/76 (100%)	*Erwinia amylovora* (pEL60)	NP_943230
76	58875-59111	→	78	hypothetical protein	78/78 (100%)	*Erwinia amylovora* (pEL60)	NP_943231
77	59196-59606	→	136	hypothetical protein	136/136 (100%)	*Erwinia amylovora* (pEL60)	NP_943232
78	59668-59973	→	101	hypothetical protein	101/101 (100%)	*Klebsiella pneumoniae (*pOXA-48a)	YP_006958818
79	60174-60614	→	146	putative anti-restriction protein KlcA	146/146 (100%)	*Klebsiella oxytoca* (pACM1)	YP_009062622
80	60658-60942	→	94	hypothetical protein	92/94 (98%)	*Citrobacter freundii* (pCTX-M3)	NP_775004
81	61097-61318	→	73	hypothetical protein	73/73 (100%)	*Serratia marcescens* (R830b)	YP_006964599
82	61378-61824	→	148	single-stranded DNA binding protein	148/148 (100%)	*Klebsiella oxytoca* (pACM1)	YP_009062621
83	61880-62191	→	103	hypothetical protein	103/103 (100%)	*Klebsiella pneumoniae* (pOXA-48a)	YP_006958823
84	62324-62860	→	178	hypothetical protein	178/178 (100%)	*Erwinia amylovora* (pEL60)	NP_943238
85	63361-63756	←	131	mobilization protein MobC	131/131 (100%)	*Klebsiella oxytoca* (pACM1)	YP_009062619
86	64001-64318	→	105	mobilization protein MobB/NikA	105/105 (100%)	*Erwinia amylovora* (pEL60)	NP_943240
87	64305-66284	→	659	mobilization protein MobA	658/659 (99%)	*Klebsiella pneumoniae* (pKPoxa-48N1)	AGL12941
88	66298-66798	→	166	conjugation protein TraH	166/166 (100%)	*Erwinia amylovora* (pEL60)	NP_943242
89	66795-67574	→	259	conjugation protein TraI	259/259 (100%)	*Citrobacter freundii* (pCTX-M3)	NP_775013
90	67585-68748	→	387	plasmid transfer ATPase TraJ	387/387 (100%)	*Citrobacter freundii* (pCTX-M3)	NP_775014
91	68738-68998	→	86	conjugation protein TraK	86/86 (100%)	*Citrobacter freundii* (pCTX-M3)	NP_775015
92	69023-72325	→	1100	primase	1100/1100 (100%)	*Klebsiella oxytoca* (pACM1)	YP_009062613
93	72249-72803	→	184	conjugation protein TraL	184/184 (100%)	*Erwinia amylovora* (pEL60)	NP_943246
94	72754-73419	→	221	hypothetical protein	221/221 (100%)	*Klebsiella oxytoca* (pACM1)	YP_009062611
95	73397-74179	→	260	conjugation protein TraM	260/260 (100%)	*Klebsiella oxytoca* (pACM1)	YP_009062610
96	74188-75339	→	383	conjugation protein TraN	383/383 (100%)	*Klebsiella oxytoca* (pACM1)	YP_009062609
97	75351-76700	→	449	conjugation protein TraO	449/449 (100%)	*Klebsiella oxytoca* (pACM1)	YP_009062608
98	76712-77416	→	234	conjugation protein TraP	234/234 (100%)	*Klebsiella oxytoca* (pACM1)	YP_009062607
99	77440-77970	→	176	conjugation protein TraQ	176/176 (100%)	*Klebsiella oxytoca* (pACM1)	YP_009062606
100	77987-78376	→	129	conjugation protein TraR	129/129 (100%)	*Klebsiella oxytoca* (pACM1)	YP_009062605
101	78422-78916	→	164	hypothetical protein	164/164 (100%)	*Citrobacter freundii* (pCTX-M3)	NP_775026
102	78913-81963	→	1016	conjugation protein TraU	1016/1016 (100%)	*Klebsiella oxytoca* (pACM1)	YP_009062604
103	81960-83168	→	402	conjugation protein TraW	402/402 (100%)	*Klebsiella oxytoca* (pACM1)	YP_009062603
104	83165-83815	→	216	conjugation protein TraX	216/216 (100%)	*Klebsiella oxytoca* (pACM1)	YP_009062602
105	83808-85988	→	726	conjugation protein TraY	726/726 (100%)	*Citrobacter freundii* (pCTX-M3)	NP_775030

Gene no.	Coding region (bp)	Strand	Protein size (aa)	Possible function	Best BLAST hits		
					aa identity (%)	Organism (plasmid)	GenBank accession no.
106	85991-86644	→	217	exclusion protein ExcA	217/217 (100%)	*Klebsiella oxytoca* (pACM1)	YP_009062600
107	86718-86948	→	76	replication protein RepC	76/76 (100%)	*Klebsiella oxytoca* (pACM1)	YP_009062599

Plasmid pIGT15 (74,839 bp)

1	297-1352	→	351	replication initiator protein, RepA	351/351 (100%)	*Enterobacter cloacae* (pENT-e56)	AIX57116
2	2725-4812	←	695	conjugation protein, TrbC	695/695 (100%)	*Serratia marcescens* (R830b)	YP_006964547
3	4825-5844	←	339	conjugation protein, TrbB	339/339 (100%)	*Klebsiella oxytoca* (pACM1)	YP_009062595
4	5786-7093	←	435	conjugation protein, TrbA	435/435 (100%)	*Klebsiella oxytoca* (pACM1)	YP_009062594
5	7093-7488	←	131	conjugation protein, TrbN	131/131 (100%)	*Klebsiella oxytoca* (pACM1)	YP_009062593
6	7593-7988	←	131	hypothetical protein	131/131 (100%)	*Klebsiella oxytoca* (pACM1)	YP_009062592
7	8056-8709	→	217	transfer inhibition protein, Tir	217/217 (100%)	*Klebsiella oxytoca* (pACM1)	YP_009062591
8	8862-9296	→	144	error-prone repair protein, MucA	144/144 (100%)	*Klebsiella oxytoca* (pACM1)	YP_009062590
9	9245-10090	→	281	translesion synthesis protein, MucB, truncated	280/281 (99%)	*Escherichia coli* HVH 87	EQP96765
10	10279-11676	←	465	methyl-accepting chemotaxis protein	465/465 (100%)	*Escherichia coli* (p1658/97)	NP_862996
11	11849-12553	←	234	IS*26* transposase	234/234 (100%)	*Klebsiella pneumoniae* (pNDM-OM)	AFZ84500
12	12578-13495	→	305	galactoside permease	305/305 (100%)	*Escherichia coli* (p1658/97)	NP_863016
13	13526-14614	←	362	recombination protein, RecF	362/362 (100%)	*Klebsiella oxytoca* (pACM1)	YP_009062589
14	14701-14982	←	93	putative K+ transporting ATPase, KdpC subunit	93/93 (100%)	Klebsiella pneumoniae subsp. pneumoniae KpQ3	EKF78970
15	15259-1619	→	286	SHV-5 beta-lactamase	286/286 (100%)	*Klebsiella oxytoca* (pACM1)	YP_009062588
16	16140-16901	←	253	DeoR family transcriptional regulator	253/253 (100%)	*Providencia stuartii* (pTC2)	YP_006965583
17	17162-18064	→	300	putative dehydrogenase	300/300 (100%)	*Klebsiella oxytoca* (pACM1)	YP_009062586
18	18076-18723	→	215	putative ygbK protein	210/210 (100%)	*Klebsiella oxytoca* (pACM1)	AAL89460.2
19	18770-19474	→	234	IS*26* transposase	234/234 (100%)	*Klebsiella pneumoniae* (pNDM-OM)	AFZ84500
20	19616-20629	←	337	integrase	337/337 (100%)	*Escherichia coli* (p1658/97)	NP_863006
21	20845-21363	→	172	aminoglycoside 6'-N-acetyltransferase	172/172 (100%)	*Klebsiella oxytoca* (pACM1)	AAC77498
22	21439-21903	→	154	aminoglycoside 3'-acetyltransferase	154/154 (100%)	*Klebsiella oxytoca* (pACM1)	YP_009062574
23	22022-22534	→	170	GCN5 family acetyltransferase	170/170 (100%)	*Klebsiella oxytoca* (pACM1)	YP_009062573
24	22550-22855	→	101	hypothetical protein	101/101 (100%)	*Klebsiella oxytoca* (pACM1)	AAC77502
25	22947-23738	→	263	3''-O-adenyltransferase	263/263 (100%)	*Klebsiella oxytoca* (pACM1)	YP_009062571
26	23902-24249	→	115	QacE-delta1	115/115 (100%)	*Klebsiella pneumoniae* (pFOX-7a)	YP_009067913
27	24243-25082	→	279	dihydropteroate synthase type I	279/279 (100%)	*Klebsiella oxytoca* (pACM1)	AIM47853
28	25210-25710	→	166	YbbA	166/166 (100%)	*Shigella flexneri 2b* (R100)	NP_052894
29	25679-26188	←	169	NTP-binding protein	169/169 (100%)	*Acinetobacter baumannii* AB0057	YP_002317676
30	26187-26981	→	264	IS*6100* transposase	264/264 (100%)	*Trueperella pyogenes*	NP_940743
31	27222-27431	←	70	Urf2Y protein	70/70 (100%)	*Klebsiella oxytoca* (pACM1)	YP_009062566
32	27497-27733	←	78	mercury resistance protein, MerE	78/78 (100%)	*Serratia marcescens* (R478)	NP_941189
33	27730-28095	←	121	transcriptional regulator MerD	121/121 (100%)	*Serratia marcescens* (R478)	NP_941190.2

Gene no.	Coding region (bp)	Strand	Protein size (aa)	Possible function	Best BLAST hits		
					aa identity (%)	Organism (plasmid)	GenBank accession no.
34	28113-29798	←	561	mercuric reductase, MerA	561/561 (100%)	*Serratia marcescens* (R478)	NP_941191
35	29837-30262	←	141	mercury transport protein, MerC	141/141 (100%)	*Serratia marcescens* (R478)	NP_941192
36	30290-30565	←	91	mercuric transport protein periplasmic component, MerP	91/91 (100%)	*Serratia marcescens* (R478)	NP_941193
37	30581-30946	←	121	mercuric transport protein, MerT	121/121 (100%)	*Serratia marcescens* (R478)	NP_941194
38	31018-31473	→	151	transcriptional regulator, MerR	151/151 (100%)	*Serratia marcescens* (R478)	NP_941195
39	31885-32562	←	225	tetracycline resistance repressor protein, TetR	225/225 (100%)	uncultured bacterium (pRSB101)	YP_133837
40	32566-33840	→	424	tetracycline resistance protein, TetA	424/424 (100%)	uncultured bacterium (pRSB201)	YP_009064288
41	33872-34756	←	294	PecM-like protein	294/294 (100%)	uncultured bacterium (pTB11)	YP_112360
42	35245-37041	→	598	truncated Tn3 transposase	598/598 (100%)	*Klebsiella oxytoca* (pACM1)	AIM47840
43	37102-37536	→	145	translesion synthesis protein, MucB, truncated	145/145 (100%)	*Klebsiella pneumoniae* (pFOX-7a)	YP_009067897
44	37562-38410	←	282	exonuclease	282/282 (100%)	*Klebsiella oxytoca* (pACM1)	YP_009062646
45	38413-38733	←	106	hypothetical protein	106/106 (100%)	*Klebsiella oxytoca* (pACM1)	YP_009062645
46	38878-39516	←	212	hypothetical protein	212/212 (100%)	*Klebsiella oxytoca* (pACM1)	YP_009062644
47	39585-39794	←	69	hypothetical protein	69/69 (100%)	*Citrobacter freundii* (pCTX-M3)	NP_774971
48	39797-40015	←	72	hypothetical protein	72/72 (100%)	*Citrobacter freundii* (pCTX-M3)	NP_774972
49	40060-40743	←	227	hypothetical protein	227/227 (100%)	*Klebsiella oxytoca* (pACM1)	YP_009062643
50	40740-41405	←	219	RelB antitoxin domain-containing protein	219/219 (100%)	*Klebsiella oxytoca* (pACM1)	YP_009062642
51	41902-42207	→	101	hypothetical protein	89/100 (89%)	*Erwinia amylovora* (pEL60)	NP_943209
52	42500-43084	→	194	hypothetical protein	194/194 (100%)	*Klebsiella oxytoca* (pACM1)	YP_009062641
53	43081-43800	→	239	hypothetical protein	239/239 (100%)	*Klebsiella oxytoca* (pACM1)	YP_009062640
54	43961-44509	←	182	hypothetical protein	182/182 (100%)	*Klebsiella oxytoca* (pACM1)	YP_009062639
55	44650-45111	→	153	hypothetical protein	153/153 (100%)	*Klebsiella oxytoca* (pACM1)	YP_009062638
56	45349-45936	→	195	hypothetical protein	195/195 (100%)	*Klebsiella oxytoca* (pACM1)	YP_009062637
57	45933-46418	→	161	hypothetical protein	161/161 (100%)	*Klebsiella oxytoca* (pACM1)	YP_009062636
58	46682-47422	→	246	resolvase	246/246 (100%)	*Erwinia amylovora* (pEL60)	NP_943215
59	47663-48637	→	324	plasmid segregation protein ParM	324/324 (100%)	*Erwinia amylovora* (pEL60)	NP_943216
60	48640-49083	→	147	plasmid stability protein B	147/147 (100%)	*Erwinia amylovora* (pEL60)	NP_943217
61	49093-49644	→	183	endonuclease Nuc	183/183 (100%)	*Erwinia amylovora* (pEL60)	NP_943218
62	49762-50268	→	168	hypothetical protein	168/168 (100%)	*Erwinia amylovora* (pEL60)	NP_943219
63	50261-50740	→	159	hypothetical protein	159/159 (100%)	*Erwinia amylovora* (pEL60)	NP_943220
64	50769-51179	→	136	hypothetical protein	136/136 (100%)	*Serratia marcescens* (R830b)	YP_006964582
65	51297-51560	→	87	hypothetical protein	87/87 (100%)	*Citrobacter freundii* (pCTX-M3)	NP_774992
66	51582-51875	→	97	hypothetical protein	79/101 (78%)	*Citrobacter freundii* (pCTX-M3)	NP_774993
67	51892-52209	→	105	mobilization protein MobB/NikA	105/105 (100%)	*Erwinia amylovora* (pEL60)	NP_943240
68	52196-54175	→	659	mobilization protein MobA	658/659 (99%)	*Klebsiella pneumoniae* (pKPoxa-48N1)	AGL12941
69	54189-54689	→	166	conjugation protein TraH	166/166 (100%)	*Erwinia amylovora* (pEL60)	NP_943242

Gene no.	Coding region (bp)	Strand	Protein size (aa)	Possible function	Best BLAST hits		
					aa identity (%)	Organism (plasmid)	GenBank accession no.
70	54686-55465	→	259	conjugation protein TraI	259/259 (100%)	*Citrobacter freundii* (pCTX-M3)	NP_775013
71	55476-56639	→	387	conjugation protein TraJ	387/387 (100%)	*Citrobacter freundii* (pCTX-M3)	NP_775014
72	56629-56889	→	86	conjugation protein TraK	86/86 (100%)	*Citrobacter freundii* (pCTX-M3)	NP_775015
73	56914-60216	→	1100	primase	1100/1100 (100%)	*Klebsiella oxytoca* (pACM1)	YP_009062613
74	60140-60694	→	184	conjugation protein TraL	184/184 (100%)	*Erwinia amylovora* (pEL60)	NP_943246
75	60660-61310	→	221	hypothetical protein	221/221 (100%)	*Klebsiella oxytoca* (pACM1)	YP_009062611
76	61288-62070	→	260	conjugation protein TraM	260/260 (100%)	*Klebsiella oxytoca* (pACM1)	YP_009062610
77	62079-63230	→	383	conjugation protein TraN	383/383 (100%)	*Klebsiella oxytoca* (pACM1)	YP_009062609
78	63242-64591	→	449	conjugation protein TraO	449/449 (100%)	*Klebsiella oxytoca* (pACM1)	YP_009062608
79	64603-65307	→	234	conjugation protein TraP	234/234 (100%)	*Klebsiella oxytoca* (pACM1)	YP_009062607
80	65331-65861	→	176	conjugation protein TraQ	176/176 (100%)	*Klebsiella oxytoca* (pACM1)	YP_009062606
81	65878-66267	→	129	conjugation protein TraR	129/129 (100%)	*Klebsiella oxytoca* (pACM1)	YP_009062605
82	66313-66807	→	164	hypothetical protein	164/164 (100%)	*Citrobacter freundii* (pCTX-M3)	NP_775026
83	66804-69854	→	1016	conjugation protein TraU	1016/1016 (100%)	*Klebsiella oxytoca* (pACM1)	YP_009062604
84	69851-71059	→	402	conjugation protein TraW	402/402 (100%)	*Klebsiella oxytoca* (pACM1)	YP_009062603
85	71056-71706	→	216	conjugation protein TraX	216/216 (100%)	*Klebsiella oxytoca* (pACM1)	YP_009062602
86	71699-73879	→	726	conjugation protein TraY	726/726 (100%)	*Citrobacter freundii* (pCTX-M3)	NP_775030
87	73882-74535	→	217	exclusion protein ExcA	217/217 (100%)	*Klebsiella oxytoca* (pACM1)	YP_009062600
88	74609-74839	→	76	replication protein, RepC	76/76 (100%)	*Klebsiella oxytoca* (pACM1)	YP_009062599
